# Human-centered design for smart home technologies: a framework for aging and mental health

**DOI:** 10.3389/fdgth.2025.1555569

**Published:** 2025-05-29

**Authors:** Mohammad Mahdi Fakhimi, Adriana Hughes, Allison M. Gustavson

**Affiliations:** ^1^ Department of Architecture, Landscape Architecture, and Interior Design, University of Minnesota, Minneapolis, MN, United States; ^2^ Department of Psychiatry, Affiliated Faculty, Rehabilitation Science Graduate Program, University of Minnesota, Minneapolis, MN, United States; ^3^ Veterans Affairs Health Systems Research Center for Care Delivery and Outcomes Research, Minneapolis VA Health Care System, Minneapolis, MN, United States; ^4^ Veterans Affairs Rehabilitation Research and Development Center for Rehabilitation & Engineering Center for Optimizing Veteran Engagement & Reintegration, Minneapolis VA Health Care System, Minneapolis, MN, United States; ^5^ Department of Medicine, University of Minnesota, Minneapolis, MN, United States

**Keywords:** human-centered design (HCD), smart home technologies, aging - old age - seniors, mental health, usability, affordability and access

## Abstract

Smart home technologies (SHTs) offer promising ways to support older adults with both mobility challenges and mental health needs, yet high costs, complex interfaces, and uncertain data practices often limit adoption. This paper addresses these challenges by proposing a human-centered design (HCD) framework focused on affordability, inclusive design for physical and cognitive variations, and transparent data governance. Through illustrative examples of low-cost sensor networks and culturally tailored voice interfaces, we argue that thoughtfully designed SHTs can promote independent living, strengthen mental health interventions, and foster user trust. We conclude by highlighting policy incentives and cross-sector collaboration as critical levers for making SHTs an accessible, sustainable tool for aging populations.

## Introduction

The rapid growth of the older adult population, projected to exceed 2.1 billion by 2050 (1), has intensified the search for sustainable solutions enabling individuals to age in place. Many older adults face mobility limitations and mental health conditions like depression and anxiety, underscoring the need for holistic home-based support (2, 3). Smart home technologies (SHTs), which integrate sensors, telehealth, and artificial intelligence, promise to address some of these challenges (4, 5). However, the reality is that SHT adoption often remains stunted by affordability barriers, interface complexity, and trust issues—especially for those with limited financial resources or cognitive impairments (6–8).

Despite evolving efforts to design SHTs, there is a persistent gap in addressing both financial accessibility and mental health integration for diverse subpopulations of older adults. This paper proposes a human-centered design (HCD) framework to bridge that gap, emphasizing cost-effective sensor networks, mobility-friendly interfaces, and integrated mental health features. We ask, “How can human-centered, affordable SHTs be deployed to support the multifaceted needs of aging populations—particularly regarding mobility and mental well-being?” Our objective is to offer both conceptual insights and practical recommendations for SHT developers, policymakers, and healthcare providers.

## Usability and accessibility barriers

### Complex interfaces and cognitive overload

Although SHTs promise daily support, many feature intricate layouts that overwhelm older adults (8). Elements such as small text, multi-level menus, and a lack of guided feedback can cause cognitive overload, particularly among those with mild impairments (9). In addition, visual, auditory, or motor-skill limitations can further complicate device interaction (10). Research consistently points to user-centered design—large fonts, simplified navigation, and voice-based commands—as a way to enhance acceptability and user experience (11).

### Mobility challenges

Physical constraints—such as reduced strength, fine motor control, or balance—pose another hurdle for older adults (12). Routine tasks, including entering passcodes or accessing devices on high shelves, can be daunting. Hardware featuring larger buttons, ergonomic grips, or sensor layouts that detect gait changes addresses these challenges directly (13). Critically, such design features also generate mobility data that may help healthcare professionals spot early warning signs of functional decline.

### Affordability and infrastructure

Financial constraints are a significant obstacle for older adults on limited incomes (14). SHT packages often involve expensive sensors, subscription fees, and the need for reliable internet access—frequently lacking in rural areas (15). Without financial support or reimbursement options, many older adults forgo these technologies, despite their potential to lower long-term healthcare costs (16). Policymakers, insurers, and tech developers must collaborate to provide affordable, user-friendly solutions that address both economic and infrastructure barriers (17).

### Privacy concerns and data silos

Continuous monitoring of mobility and emotional states often raises concerns about surveillance and data misuse (18). Older adults may reject “always-on” systems if data policies are unclear or if platforms fail to integrate, resulting in fragmented records (19). Such silos hinder early interventions, like detecting depression risks from mobility or sleep pattern changes. Experts recommend robust encryption, transparent consent processes, and standardized protocols to unify data while safeguarding user privacy (20).

### Additional insights on usability and trust

Recent studies underscore the importance of real-time monitoring features that respect user privacy while enabling proactive interventions (21). Likewise, a user-centered, co-design approach can mitigate adoption barriers by involving older adults directly in the development process (22). Finally, ensuring a pleasurable user experience—from intuitive navigation to transparent data-sharing policies—can build trust and reduce fears of misuse among aging populations (23). By integrating these best practices alongside the strategies discussed above, SHTs can become genuinely accessible, affordable, and empowering solutions for diverse older-adult communities.

## Potential of affordable mobility-enhancing SHT solutions

### Low-cost sensor networks

Open-source sensor platforms offer affordable solutions for tracking daily activities, detecting falls, and monitoring behavioral changes, such as reduced cooking or socializing (21). These cost-effective systems provide continuous data streams that support mental health assessments (24). By linking mobility patterns to emotional well-being, healthcare providers can intervene early to address risks of depression or cognitive decline (4, 5).

### Adaptive interfaces for varying abilities

Voice-activated assistants, gesture-based navigation, and simplified screens enhance accessibility (22). Individuals with arthritis or tremors benefit from larger on-screen icons and fewer steps (12). Wearable gadgets (e.g., smartwatches with oversized symbols) paired with in-home sensors ensure around-the-clock coverage—even if users are away from the central console (25). Telehealth integration allows for remote mental health or physical therapy check-ins (26).

### Community-oriented mobility support

Aging in place is not confined to one's residence. Linking SHTs to community resources—like ride-share services or tele-rehabilitation—helps older adults stay connected (23). Location tracking and user-friendly scheduling apps can guide older individuals who might need reminders or real-time navigation. Alleviating social isolation also mitigates depression and encourages sustained participation in daily life (3).

### Policy incentives for affordability

Home-based solutions can reduce institutional care expenses, prompting some policymakers and insurers to explore subsidies for broadband or SHT hardware (17). Lowering upfront costs is key to expanding adoption (15). Incentives such as tax credits or reimbursement models tied to improved health outcomes can also encourage developers to address older users' unique needs. Together, these strategies can transition SHTs from niche products to widely accessible tools (16).

## Human-centered design: a core framework

### Co-creation and iterative feedback

Genuine older-adult participation in all design stages—from concept to testing—helps reveal otherwise overlooked usability issues (27). Cultural, linguistic, and cognitive variations come to light through user workshops and pilot studies. Iterative cycles also capture shifting health or mobility needs, ensuring that SHTs remain relevant (26).

### Personalization and cultural alignment

Adapting SHTs to local language, customs, or personal preferences promotes ongoing engagement (28). For example, daily mood-check prompts in a user's native tongue or optional privacy settings for motion sensors can mitigate discomfort and stigma. Flexible settings (e.g., customizable voice pitch or text size) address diverse needs and reflect a commitment to inclusive design (29).

### Data transparency and security

Older adults often have heightened concerns about data-driven technologies (18). Providing clear dashboards that show active sensors and offering granular consent builds trust (20). Encryption and user choice in data sharing further bolster autonomy—key elements for mental health support (19, 30).

### Holistic monitoring of physical and mental health

Integrating multiple data sources—like heart rate, gait, and mood logs—creates a fuller portrait of well-being (5, 9). For instance, detecting a sudden dip in physical activity alongside reported low mood might signal an impending depressive episode (6). By securely sharing insights with authorized caregivers or clinicians, interventions can be proactive rather than crisis-driven (13, 16).

## Discussion

**Figure 1:**
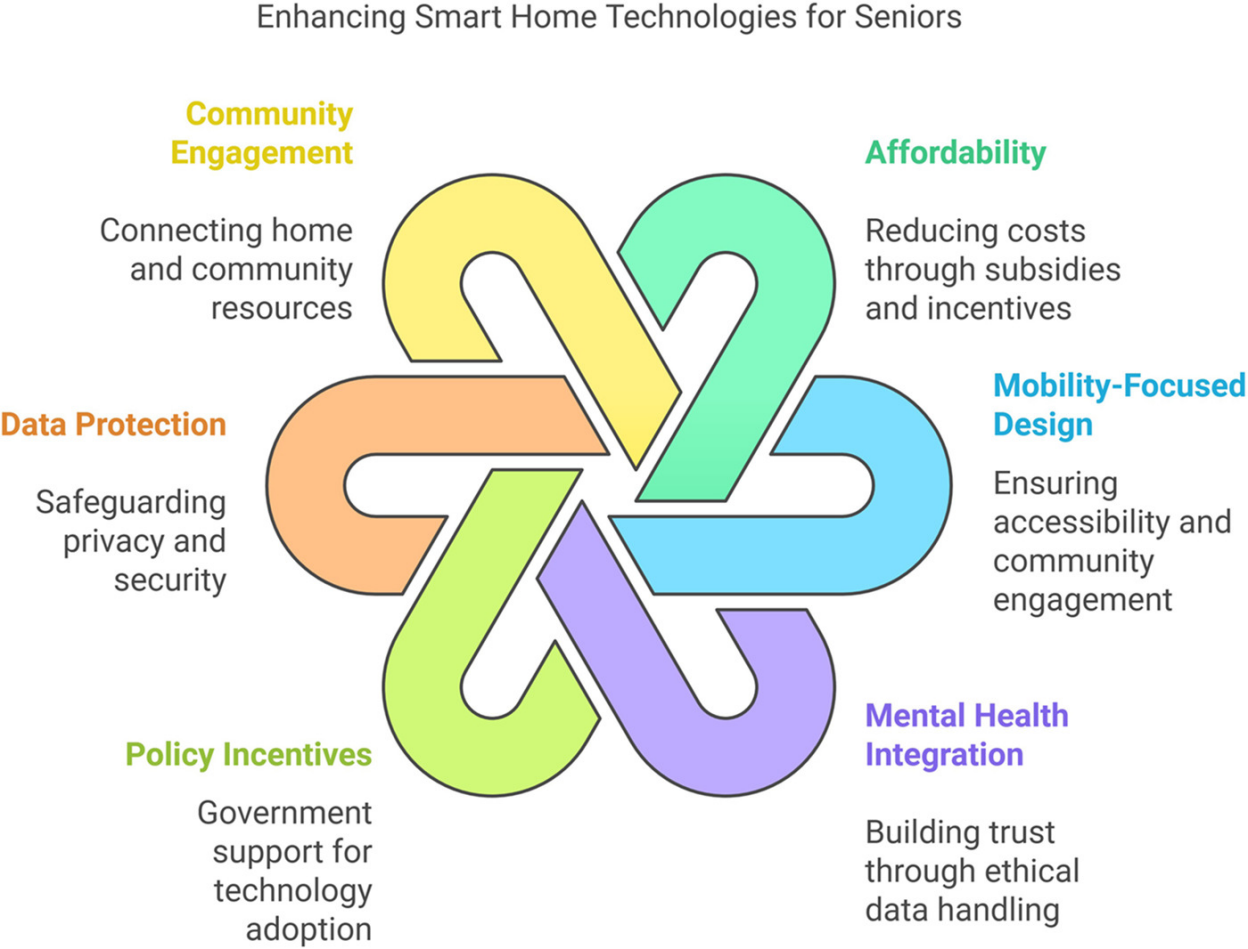
HCD for smart home technologies in aging in place.

As illustrated in Figure 1, affordability and mobility are essential for making SHTs accessible to older adults. While well-designed systems can detect or delay cognitive decline (4, 6), barriers like cost and privacy concerns persist (15, 18). Collaborative efforts among policymakers, technologists, and healthcare providers are necessary to develop cost-effective hardware, intuitive interfaces, and robust privacy safeguards.

### Limitations

While our proposed framework highlights affordability, user-centered design, and mental health support, further empirical studies are needed to test these integrated approaches in large-scale, long-term deployments. Continuous updates and maintenance costs could also pose a barrier for low-income users or under-resourced communities. Additionally, privacy concerns remain an ongoing challenge; older adults may still feel uneasy about sensors or voice assistants tracking their activities.

### Key recommendations

1. **Subsidies and Incentives:** Financial support from governments or insurers can lower initial costs and boost adoption (17).
2. **Collaborative Design:** Involve older adults, caregivers, healthcare professionals, and technologists in iterative co-creation processes (27).
3. **Transparent Data Practices:** Clear policies and user-friendly privacy dashboards can build trust and encourage engagement (19).
4. **Community Integration:** Linking SHTs to local transit, social activities, and telehealth services supports holistic well-being (3, 26).

## Conclusion

By centering on affordability, usability, and mental health integration, the proposed HCD framework offers a pathway to make SHTs more inclusive and effective for diverse aging populations. Although challenges persist—such as ensuring user privacy and identifying sustainable financing models—our real-world examples suggest that well-planned, low-cost solutions can improve both mobility and mental health outcomes. Continued collaboration among policymakers, developers, and healthcare providers is essential to refine SHT designs and expand access, ultimately empowering older adults to age with dignity and independence.
